# Real-world evaluation of a computed tomography-first triage strategy for suspected Coronavirus disease 2019 in outpatients in Japan: An observational cohort study

**DOI:** 10.1097/MD.0000000000026161

**Published:** 2021-06-04

**Authors:** Shigeta Miyake, Takuma Higurashi, Takashi Jono, Taisuke Akimoto, Fumihiro Ogawa, Yasufumi Oi, Katsushi Tanaka, Yu Hara, Nobuaki Kobayashi, Hideaki Kato, Tsuneo Yamashiro, Daisuke Utsunomiya, Atsushi Nakajima, Tetsuya Yamamoto, Shin Maeda, Takeshi Kaneko, Ichiro Takeuchi

**Affiliations:** a Team COVID-19, Yokohama City University Hospital; b Department of Neurosurgery; c Department of Gastroenterology and Hepatology; d Department of Neurology and Stroke Medicine; e Department of Emergency Medicine; f Department of Pulmonology, Yokohama City University Graduate School of Medicine, 3-9 Fukuura, Kanazawa, Yokohama; g Infection Prevention and Control Department, Yokohama City University Hospital, 3-9 Fukuura, Kanazawa-ku; h Department of Diagnostic Radiology, Yokohama City University Graduate School of Medicine, 3-9, Fukuura, Kanazawa, Yokohama, Kanagawa; i Department of Gastroenterology, Yokohama City University Graduate School of Medicine, 3-9 Fukuura, Kanazawa, Yokohama, Japan.

**Keywords:** computed tomography, computed tomography-first triage, Coronavirus disease 2019, diagnosis, emergency treatment, fever, pneumonia

## Abstract

The Coronavirus disease 2019 pandemic continues to spread worldwide. Because of the absence of reliable rapid diagnostic systems, patients with symptoms of Coronavirus disease 2019 are treated as suspected of the disease. Use of computed tomography findings in Coronavirus disease 2019 are expected to be a reasonable method for triaging patients, and computed tomography-first triage strategies have been proposed. However, clinical evaluation of a computed tomography-first triage protocol is lacking.

The aim of this study is to investigate the real-world efficacy and limitations of a computed tomography-first triage strategy in patients with suspected Coronavirus disease 2019.

This was a single-center cohort study evaluating outpatients with fever who received medical examination at Yokohama City University Hospital, prospectively registered between 9 February and 5 May 2020. We treated according to the computed tomography-first triage protocol. The primary outcome was efficacy of the computed tomography-first triage protocol for patients with fever in an outpatient clinic. Efficacy of the computed tomography-first triage protocol for outpatients with fever was evaluated using sensitivity, specificity, positive predictive value, and negative predictive value. We conducted additional analyses of the isolation time of feverish outpatients and final diagnoses.

In total, 108 consecutive outpatients with fever were examined at our hospital. Using the computed tomography-first triage protocol, 48 (44.9%) patients were classified as suspected Coronavirus disease 2019. Nine patients (18.8%) in this group were positive for severe acute respiratory syndrome coronavirus 2 using polymerase chain reaction; no patients in the group considered less likely to have Coronavirus disease 2019 tested positive for the virus. The protocol significantly shortened the duration of isolation for the not-suspected versus the suspected group (70.5 vs 1037.0 minutes, *P* < .001).

Our computed tomography-first triage protocol was acceptable for screening patients with suspected Coronavirus disease 2019. This protocol will be helpful for appropriate triage, especially in areas where polymerase chain reaction is inadequate.

## 1 Introduction

The pandemic of Coronavirus disease 2019 (COVID-19) is currently one of the greatest human challenges for health care systems and economic systems worldwide.^[1–3]^ COVID-19, caused by severe acute respiratory syndrome coronavirus 2 (SARS-CoV-2), has affected more than 4.6 million individuals, and has caused over 300,000 deaths to date.^[4]^ The severity and case fatality rate of COVID-19 differs from country to country, depending on the health care system and health policies.^[5,6]^ In general, the medical collapse, caused by surpassing the health care capacity for the intensive care patients, may trigger an increase in the death rate.

Several studies have revealed factors associated with poor prognosis such as older age, underlying comorbidities, clinical symptoms, and hematology findings.^[7]^ Because hospitalized patients constitute a high-risk population, hospital outbreaks are among the most dangerous situations. Therefore, adequate use of disposable personal protective equipment (PPE) is recommended.^[8]^ Because of a global shortage of PPE, there are many reports of hospital outbreaks of COVID-19 and infection of medical staff. To prevent such outbreaks in hospitals, it is advisable to separate services for patients with suspected COVID-19.^[5]^

To reduce consumption of PPE and implement appropriate isolation, triage to distinguish patients who are less likely to have COVID-19 from those with suspected COVID-19 is necessary.^[9,10]^ However, there are no established protocols for managing patients with suspected COVID-19 who present to the emergency department. Because reliable rapid diagnostic systems are lacking, patients with symptoms related to COVID-19 such as fever, fatigue, respiratory symptoms, headache, and taste or olfactory disorders are widely treated as suspected COVID-19. Several triage protocols and their efficiency have been reported.^[9,11,12]^ However, because COVID-19 symptoms are not specific to that disease, there might be many differential diagnoses in patients with suspected COVID-19. Recently, radiological findings of COVID-19 have been reported.^[12–14]^ Generally, chest X-ray is useful to diagnose pneumonia; however, it is unsuitable for COVID-19. Because the characteristics of chest radiological images in COVID-19 mainly appear in the interstitium, revealing ground-glass opacities (GGO), it is difficult to diagnose COVID-19 using chest X-ray alone, especially in mild cases. Presently, computed tomography (CT) in patients with COVID-19 can be considered a reasonable method for triaging patients with COVID-19. However, consensus has not been reached regarding clinical evaluation of triage protocols using CT.^[15]^

COVID-19 has affected 17,018 individuals and caused 903 deaths in Japan as of 3 June, 2020.^[16]^ Yokohama City is the second largest city in Japan. Since February 2020, we have treated patients with COVID-19 at Yokohama City University Hospital, a tertiary-level hospital, owing to the outbreak on the “Diamond Princess” cruise ship docked in Yokohama Bay.^[17,18]^ A select team (Team COVID-19) was deployed for the treatment of patients with moderate to severe COVID-19 and with suspected COVID-19. One focus of Team COVID is on preventing in-hospital infections and medical staff infections, with reference to protocols that were previously launched during the epidemic of a novel type of influenza in 2002 to 2003. Team COVID-19 comprises 10 doctors (mainly from the internal medicine and surgery departments) and engages with the outpatient clinic and hospitalized patients with suspected COVID-19 in a 24-hour rotation schedule. At our hospital, a protocol adopting CT as the first-line examination, the “CT-first triage protocol,” has been used in the management of patients with suspected COVID-19. We adopted the CT-first triage protocol for several reasons. There are not many patients with COVID-19 in Japan; thus, polymerase chain reaction (PCR) testing of individuals with no or mild symptoms might lead to many false positive results because of low sensitivity of the PCR test.^[19,20]^ Furthermore, in some regions, such as Wuhan and European countries, many people with mild symptoms rushed to a hospital to receive PCR testing, leading to the collapse of medical services and subsequently, many in-hospital infections. To avoid such events and keep hospitals functioning normally, adopting the CT-first triage protocol was reasonable in Japan. To verify the efficacy and limitations of this protocol, we focused on the characteristics of outpatients with fever and results using the CT-first triage protocol. To our knowledge, this is the first detailed report using real-world clinical data regarding a CT-first triage protocol used for patients with suspected COVID-19.

## 2 Methods

### 2.1 Study design and setting

This was a single-center cohort study aiming to evaluate outpatients with fever who received medical examination at Yokohama City University Hospital, prospectively registered between 9 February, 2020 and 5 May, 2020.

### 2.2 Ethical approval

The Institutional Review Board at Yokohama City University Hospital approved this study (approval number B200200047). Consent for participation in this study was obtained from all patients after explaining the clinical study by verbal or a description of how to opt out, because this study is prospectively registered retrospective review (https://www.yokohama-cu.ac.jp/amedrc/ethics/ethical/fuzoku_optout.html).

### 2.3 Participants

Among all patients who visited our outpatient clinic, those who exhibited COVID-19-like symptoms such as fever (over 37.5°C), fatigue, respiratory symptoms, headache, and taste or olfactory disorders, were identified and instructed to visit our separate clinic for outpatients with fever. Outpatients with fever were treated as suspected COVID-19 at this clinic, regardless of whether they had a fever during the study period.

We analyzed the following baseline patient characteristics: age, sex, history of overseas travel, contact history with a confirmed COVID-19 case, and underlying comorbidities (chronic pulmonary disease, diabetes, hypertension, chronic renal failure, cardiovascular disease, cerebrovascular disease, and malignancy). We also evaluated patients’ existing symptoms such as fever, fatigue, respiratory symptoms, headache, and taste or olfactory disorders. We or anyone else have not reported these patients in any other submission.

### 2.4 Interventions and measurements

#### 2.4.1 CT-first triage protocol

**Figure 1:**
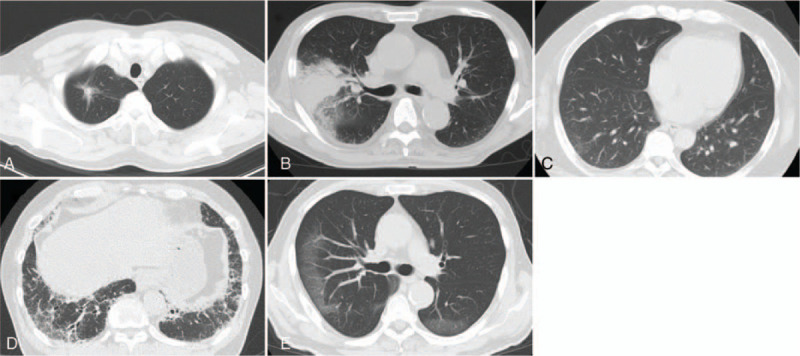
Representative features of chest computed tomography (CT) classifications. Chest CT scans of outpatients with fever. CT features classified into 5 categories according to the COVID-19 Reporting and Data System (CO-RADS). (A) CO-RADS 1, normal CT or non-infectious etiology (e.g., lung tumor, lung fibrosis). (B) CO-RADS 2, infectious etiology that is not compatible with COVID-19 (e.g., infectious bronchiolitis, bronchopneumonia, lobar pneumonia). (C) CO-RADS 3, equivocal findings for COVID-19 (e.g., homogeneous extensive ground-glass opacities [arrow]). (D) CO-RADS 4, typical CT findings of COVID-19 (similar to CO-RADS 5) but showing some overlap with other pneumonia. (E) CO-RADS 5, typical CT findings of COVID-19 (e.g., multifocal ground-glass opacities with or without consolidation in lung regions close to visceral pleural surfaces).

First, patients complaining of fever, fatigue, respiratory symptoms, headache, and taste or olfactory disorder are redirected to the separate outpatient clinic of the emergency room described above. From that time, patients are isolated from other patients and treated as suspected COVID-19. Second, a nurse of in the outpatient clinic equipped with PPE briefly checks patients’ vital signs and their present history. Then, a chest CT scan is conducted. Our hospital is equipped with 3 CT scanning suites. We set one of these as a dedicated suite for patients with suspected or confirmed COVID-19. Patients with suspected COVID-19 are separated from other patients in the transport corridors and elevators to the CT suite. We use an 80-detector-row CT scanner (Aquilion Prime, Canon Medical Systems, Japan) with 120-kVp X-ray tube voltage and automatic tube current modulation. The 2-pattern CT images are reconstructed with a slice thickness/increment of 5.0 mm/5.0 mm and 0.5 mm/0.5 mm. Coronal and sagittal multiplanar reconstruction images are also acquired. All CT findings are immediately interpreted by specialized radiologists and Team COVID-19 doctors, and classified into 5 categories according to the COVID-19 Reporting and Data System (CO-RADS).^[21]^ CO-RADS is used to assess suspected pulmonary involvement in COVID-19 on a scale from 1 (very low) to 5 (very high), as follows: CO-RADS 1, normal CT or non-infectious etiology (e.g., lung tumor, lung fibrosis); CO-RADS 2, infectious etiology that is not compatible with COVID-19 (e.g., infectious bronchiolitis, bronchopneumonia, lobar pneumonia); CO-RADS 3, equivocal findings for COVID-19 (e.g., homogeneous extensive GGO); CO-RADS 4, typical CT findings of COVID-19 (similar to CO-RADS 5) but showing some overlap with other pneumonia; CO-RADS 5, typical CT findings of COVID-19 (e.g., multifocal GGO with or without consolidation in lung regions close to visceral pleural surfaces). Representative images in each category are shown in Figure 1. Lastly, patients are separated into a “COVID-19 suspected” group or a “COVID-19 less likely” group. Patients with a suspicious clinical history (contact with an infected person and overseas travel history) or suspicious findings on chest CT scan (CO-RADS 3–5) or who were diagnosed with suspected COVID-19 by a Team COVID doctor were included in the suspected COVID-19 group. Patients with neither a suspicious clinical history nor chest CT features suggestive of COVID-19 (CO-RADS 1–2) were included in the COVID-19 less likely group. If patients were diagnosed as less likely to have COVID-19, isolation and PPE support were discontinued, and detailed physical and laboratory examinations were performed. However, if patients were diagnosed with suspected COVID-19, isolation and PPE support were continued until a negative PCR test result was obtained for the patient. A PCR test for SARS-CoV-2 was performed only in patients with suspected COVID-19. All samples obtained after 11:00 on the previous day were immediately submitted for PCR testing each day, and the results were obtained at 15:00 on the same day.

### 2.5 Outcomes

The primary outcome was the efficacy of the CT-first triage protocol in patients with suspected COVID-19. We assessed all results of PCR for SARS-CoV-2 and the final diagnoses of patients. Patients who did not undergo PCR testing were followed for up to 14 days until confirmed clinically negative for COVID-19. Secondary outcomes were the duration of isolation among all patients and safety of the medical staff and hospitalized patients. Isolation duration was defined as the time that PPE was required or that patients were isolated from medical staff and other patients to prevent COVID-19 infection. For the suspected COVID-19 group, the time to a PCR-confirmed negative result was applicable for determining the duration of isolation. The isolation duration of PCR-positive patients was excluded from the results for isolation duration in the suspected COVID-19 group because COVID-19-positive patients remained in isolation until their treatment was finished. However, in the group less likely to have COVID-19, the time to a CT-confirmed negative result was applicable to determine the isolation duration. The patient data were retrospectively examined using medical records.

### 2.6 Statistical analysis

To account for biases, we performed the analysis after the study period was complete. Results are presented as mean for quantitative data and as frequency (percentage) for categorical data. The Student's *t* test was used for continuous data. A *P* value < .05 was considered statistically significant. All statistical analyses were performed using Prism 7.9 J for Windows (GraphPad Software, Inc., San Diego, CA).

## 3 Results

### 3.1 Characteristics of outpatients with fever

Between 9 February, 2020 and 5 May, 2020, a total 108 outpatients with fever received a medical examination at our hospital, and all of them were included in this study. The mean participant age was 58.9 ± 19.5 years (range, 18–101 years), and 60 patients (55.6%) were male. Four patients had an overseas travel history and 8 patients had contact with a confirmed COVID-19 case. Nearly 70% of patients had comorbidities such as chronic pulmonary disease (21, 19.4%), hypertension (25, 23.1%), and malignancy (20, 18.5%). The most common symptom was fever (77, 71.2%) and 55 patients (50.9%) reported respiratory symptoms (Table 1).

**Table 1:** Characteristics of outpatients with fever.

Characteristics	N = 108	
Age, yr (mean ± SD, range)	58.9 ± 19.5	Range: 18–101
Sex		
Male	60	55.6%
Female	48	44.4%
Clinical history		
Overseas travel history	4	3.7%
Contact with a COVID-19 case	8	7.4%
Comorbidity		
Any	74	68.5%
Chronic pulmonary disease	21	19.4%
Diabetes	14	13.0%
Hypertension	25	23.1%
Chronic renal failure	6	5.6%
Cardiovascular disease	10	9.3%
Cerebrovascular disease	4	3.7%
Malignancy	20	18.5%
Symptoms		
Fever	77	71.2%
Respiratory symptoms	55	50.9%
Fatigue	37	34.3%
Headache	20	18.5%
Taste or olfactory disorder	7	6.5%

### 3.2 Triage results of the CT-first triage protocol

**Figure 2:**
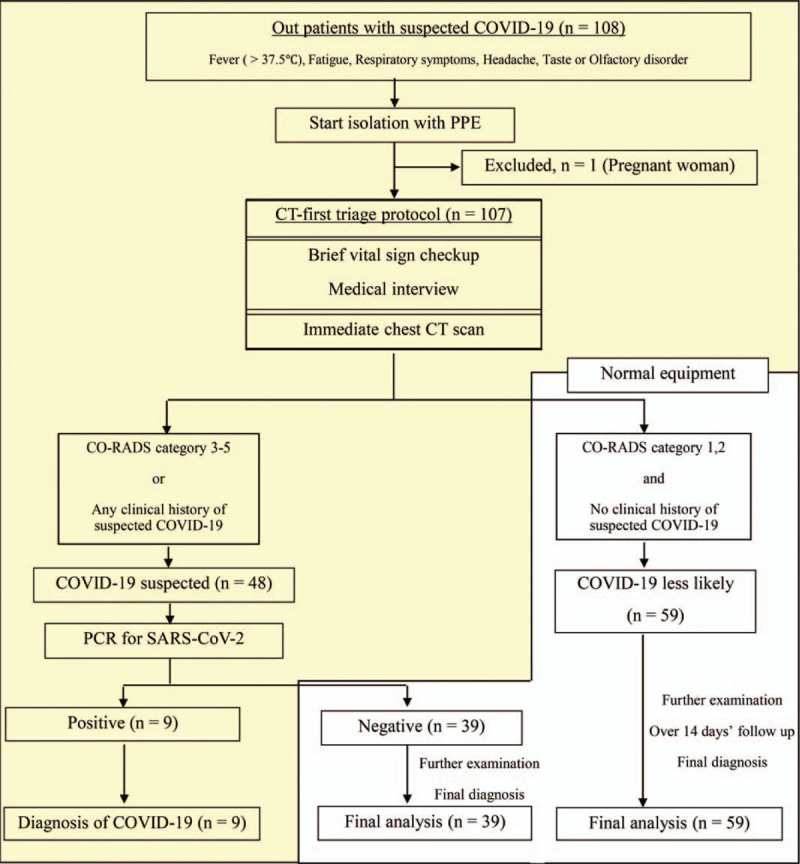
Computed tomography (CT)-first triage protocol for outpatients with fever and participant flow. Overview of the CT-first triage protocol for outpatients with fever and participant flow. Yellow area indicates duration of isolation, requiring personal protective equipment (PPE). One patient was excluded from the CT-first triage protocol because of pregnancy; the remainder were triaged using the protocol. Patients in the group less likely to have Coronavirus disease 2019 (COVID-19), classified according to the COVID-19 Reporting and Data System (CO-RADS) and the patient's clinical history, were treated using normal equipment and were finally diagnosed after further examination. The “COVID-19 less likely” group was followed for 14 days after visiting our outpatient clinic. The suspected COVID-19 group was tested using polymerase chain reaction (PCR) for severe acute respiratory syndrome coronavirus 2 (SARS-CoV-2). Patients with negative PCR test results for SARS-Co-2 were treated using normal equipment and were finally diagnosed after further examination.

In conducting the CT-first triage protocol, 1 patient refused chest CT because she was pregnant. The remaining outpatients with fever in the clinic received chest CT and all images were immediately categorized according to the CO-RADS by specialized radiologists and Team COVID-19 doctors. Forty-eight (44.9%) patients were categorized as CO-RADS 1, 26 (24.3%) CO-RADS 2, 14 (13.1%) CO-RADS 3, 6 (5.6%) CO-RADS 4, and 13 (12.1%) patients were categorized as CO-RADS 5. Finally, 48 (44.9%) patients were included in the COVID-19 suspected group. On chest CT, two-thirds of patients in the suspected COVID-19 group showed features of CO-RADS categories 3 to 5; the remaining patients in the suspected COVID-19 group were classified according to their clinical history and other examinations conducted by Team COVID-19 doctors. All patients in the COVID-19 less likely group showed features of CO-RADS categories 1 or 2 and had no clinical history suspicious for COVID-19. The participant flow is shown in Figure 2.

Isolation was continued until patients were diagnosed as either unlikely to have COVID-19 using the CT-first triage protocol or had a confirmed negative PCR test result for SARS-CoV-2. The mean duration of isolation was significantly shorter in the COVID-19 less likely group (70.5 ± 41.2 minutes) than in the group with suspected COVID-19 (1037.0 ± 512 minutes) (*P* < .001) (Table 2).

**Table 2:** Results of CT-first protocol.

	COVID-19 suspected (n = 48)		COVID-19 less likely (n = 59)		*P*
Patients	48	44.9%	59	55.1%	
Age, yr (mean ± SD, range)	61.1 ± 17.8	Range: 21–90	57.3 ± 20.9	Range: 18–101	
Sex (number, %)					
Male	27	56.3%	33	55.9%	
Female	21	43.8%	26	44.1%	
CT feature					
CO-RADS (number, %)					
Category 1	6	12.5%	42	71.2%	
Category 2	9	18.8%	17	28.8%	
Category 3	14	29.2%	0	0.0%	
Category 4	6	12.5%	0	0.0%	
Category 5	13	27.1%	0	0.0%	
Duration of isolation					
Time until CT confirmation, min (mean ± SD, range)	81.7 ± 48.8	Range: 23–289	70.5 ± 41.2	Range: 19–297	
Time until PCR confirmation, min (mean ± SD, range)	1013 ± 505.5	Range: 255–1665	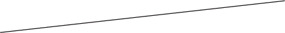		
Isolation duration, non-COVID-19 group, min (mean ± SD, range)	1037.0 ± 512	Range: 255–1665	70.5 ± 41.2	Range: 19–297	*P *< .001

### 3.3 Final clinical diagnosis after CT-first triage protocol

All patients in the suspected COVID-19 group underwent PCR testing for SARS-Co-V-2. Among 48 patients in this group, 9 (18.8%) were positive for SARS-CoV-2 on PCR and the remainder were negative. One patients with suspected COVID-19 had 1 negative PCR test result; however, Team COVID doctors strongly suspected COVID-19 because the patient was categorized as CO-RADS 5 according to CT findings; the patient was retested and definitively diagnosed with COVID-19 after 2 positive PCR test results. The others 8 of 9 patients definitely diagnosed with COVID-19 had CT features of CO-RADS category 5. However, during the study period, no patients were subsequently diagnosed with COVID-19 in the group less likely to have COVID-19. The final diagnoses of outpatients with fever who did not have COVID-19 were respiratory infection (51, 47.7%), other focal infection (22, 20.6%), tumor-related fever (5, 4.7%), and interstitial pneumonia (4, 3.7%). Seven outpatients (6.5%) had life-threatening conditions that mimicked COVID-19: 4 with meningitis, 1 with acute myocardial infarction, and 1 patient who had pneumonia with venous thrombosis. Cases of life-threatening conditions among our outpatients with fever are described in Supplemental material. A total 31 patients (64.6%) in the COVID-19 suspected group and 20 patients (33.9%) in the COVID-19 less likely group were hospitalized. One patient with suspected COVID-19 died owing to severe COVID-19 causing multiple organ failure, and 1 patient in the COVID-19 less likely group died owing to acute myocardial infarction (Table 3).

**Table 3:** Clinical outcomes.

	COVID-19 suspected (n = 48)		COVID-19 less likely (n = 59)	
Final diagnosis				
COVID-19	9	18.8%	0	0.0%
Not COVID-19	39	81.2%	59	100%
Respiratory infection	23	47.9%	28	47.5%
Other focal infection (meningitis)	7 (2)	14.6% (4.2%)	15 (2)	25.4% (3.4%)
Interstitial pneumoniae	4	8.3%	0	0.0%
Tumor-related fever	2	4.2%	3	5.1%
Collagen disease	0	0.0%	2	3.4%
Acute myocardial infarction	0	0.0%	1	1.7%
Others	3	6.3%	10	16.9%
Clinical outcome				
Death	1	2.1%	1	1.7%
Hospitalization	31	64.6%	20	33.9%
Not hospitalized	17	35.4%	39	66.1%

### 3.4 Infection control during the study period

Via separation of outpatients with fever in our specialized outpatient clinic and using the CT-first triage protocol, adequate isolation was achieved for these outpatients. Throughout the study period, no medical staff developed fever or other health concerns. In addition, no in-hospital infections occurred, and we were able to use less PPE.

## 4 Discussion

In this study, we report the evaluation results for the CT-first triage protocol in outpatients with fever suspected of having COVID-19 at our clinic. To our knowledge, these are the first real-world clinical data regarding a CT-first triage protocol worldwide. In our experience, most outpatients with fever did not have COVID-19 (Table 1). From the perspective of conserving medical resources, a reasonable triage protocol is essential. We demonstrated the effectiveness of our CT-first triage protocol in selected outpatients suspected of having COVID-19 based on their clinical history and chest CT features. In our experience, the CT-first triage protocol is acceptable for clinical use. Our CT-first triage protocol improved the pre-test probability of PCR testing for SARS-CoV-2 from 8.4% to 18.8% (Table 2). Furthermore, no medical staff or hospitalized patients developed new COVID-19-related symptoms during the study period. The consumption of PPE was also kept to a minimum.

To respond to the threat of infectious diseases, reliable rapid diagnostic technologies like that for influenza are required. The use of new technologies including artificial intelligence (AI),^[22]^ specific antibody,^[23]^ and immunochromatography^[24]^ has recently been reported. For clinical use, these new technologies clearly require additional studies and concrete evidence. Nevertheless, increasing pre-test probability using AI-based medical interviews and AI analysis of CT scans are promising directions.^[25]^ Our hospital is equipped 3 CT scanning suites and 1 is a dedicated suite for patients with suspected or confirmed COVID-19. Therefore, the CT-first triage protocol is currently feasible for screening suspected cases of COVID-19. Whereas the CO-RADS categories are reliable, owing to the existence of false-negative CT categorization,^[21]^ a protocol combining the clinical history of COVID-19 risk factors and CT features is recommended. Prokop et al reported that 13.8% (95% confidence interval 9–18%) of cases were false negatives in their cohort of 840 images.^[21]^ We used CO-RADS and patients’ clinical history for diagnosis; there were no false-negative COVID-19 cases in the group classified as less likely to have COVID-19.

One of the merits of our CT-first triage protocol is the ability to quickly release patients from isolation who are unlikely to have COVID-19 (Table 2). In general, COVID-19 was diagnosed with PCR testing for SARS-CoV-2. However, PCR tests normally require at least several hours, and the PCR test for SARS-CoV-2 diagnosis takes longer because it is performed using several specimens at once. In that sense, the CT-first protocol is an acceptable method to judge whether patients who are unlikely to have COVID-19 can be released from isolation. However, in cases of suspected COVID-19, physicians generally tend to do less thorough examinations. This is because several studies have shown that certain medical procedures carry a high risk of viral exposure for medical staff.^[26,27]^ Moreover, patients with suspected COVID-19 must be isolated from other patients owing to the risk of infection via aerosols that can remain in the air for several hours.^[28,29]^ Therefore, it is easy to fail to reduce risk or narrow the differential diagnosis and delay the start of therapy. A CT scan seems unnecessary for diagnosis in many cases, and the time lost before the start of therapy could be reduced. However, from the perspectives of protecting medical staff, conserving resources, and returning to the normal diagnostic flow, use of the CT-first triage protocol is acceptable.

We should emphasize that physicians are susceptible to cognitive bias (confirming bias) with respect to the clinical history and CT features of COVID-19.^[30,31]^ Some of our patients had life-threatening conditions that may mimic COVID-19. Because the symptoms and chest CT features of COVID-19 are non-specific, the CT-first triage protocol overestimates patients with COVID-19.^[15,32]^ As mentioned, several life-threatening conditions that mimic COVID-19 were present in our outpatients. Therefore, medical doctors must be aware that patients suspected of COVID-19 may have any one of a number of diseases. Careful assessment of vital signs and medical interviews is important, and wide differential diagnosis and checking for life-threatening diseases are crucial.

There are some limitations in our study. First, this was a retrospective review at a single center. The characteristics of our outpatients with fever may depend on the study region and hospital characteristics. Our hospital is a tertiary-level hospital in Yokohama City and the patients in our hospital had many comorbidities; therefore, patient bias may be present. Furthermore, a hospital's medical protocol depends its medical resources; future validation studies are needed to confirm our results. Second, the number of patients was limited; therefore, further studies among outpatients with fever are needed to yield more concrete evidence and confirm our findings. Finally, we did not perform SARS-CoV-2 PCR testing in the group of patients classified as less likely to have COVID-19. Thus, we might have missed COVID-19 cases in this group because many patients with COVID-19 have mild or no symptoms.^[1,33]^ However, no patients were diagnosed with COVID-19 in this group during follow-up, and there were no cases of secondary infection originating from COVID-19. Furthermore, 1 purpose of establishing an outpatient clinic for individuals presenting with fever is to separate those suspected of having COVID-19 from other patients and to protect medical staff. In that sense, the CT-first triage protocol was useful; no medical staff infections or in-hospital infections occurred during the study period. At the same time, the patients categorized with COVID-19 less likely group should continue adequate mask use and contact infection prevention for the case of false negative.

## 5 Conclusion

We demonstrated the efficacy and safety of the CT-first triage protocol for patients with suspected COVID-19 in a real-world setting. Our CT-first triage protocol is acceptable for screening patients with suspected COVID-19. Whereas the CT-first triage protocol is adequate for triage of COVID-19, there is a risk of delayed diagnosis and the start of therapy for life-threatening conditions that mimic COVID-19. Our findings might improve the accuracy of diagnosis for outpatients with fever in the present era of COVID-19 and can be helpful for appropriate triage, especially in settings where PCR testing is inadequate.

## Acknowledgments

We wish to thank all the staff at our institution for their support in COVID-19 treatment, as well as the study participants and their families. We thank Analisa Avila, ELS, of Edanz Group (https://en-author-services.edanzgroup.com/) for editing a draft of this manuscript.

## Author contributions

SM and TH conceived the study, designed the trial. TJ, TA, FO, YO, KT, YH, NK, and HK undertook recruitment of participating centers and patients and managed the data, including quality control. TY and DU conducted radiological analyses. AN, TY, SM, TK, and IT supervised the conduct of the trial and data collection. All authors contributed substantially to its revision. TH takes responsibility for the paper as a whole.

All authors attest to meeting the 4 ICMJE.org authorship criteria:

(1) Substantial contributions to the conception or design of the work; or the acquisition, analysis, or interpretation of data for the work; AND
(2) Drafting the work or revising it critically for important intellectual content; AND
(3) Final approval of the version to be published; AND
(4) Agreement to be accountable for all aspects of the work in ensuring that questions related to the accuracy or integrity of any part of the work are appropriately investigated and resolved.

**Conceptualization:** Shigeta Miyake, Takuma Higurashi.

**Data curation:** Fumihiro Ogawa, Yasufumi Oi, Hideaki Kato.

**Formal analysis:** Shigeta Miyake.

**Investigation:** Yasufumi Oi, Hideaki Kato, Tsuneo Yamashiro.

**Methodology:** Katsushi Tanaka, Yu Hara, Nobuyuki Kobayashi, Hideaki Kato, Daisuke Utsunomiya, Tetsuya Yamamoto.

**Supervision:** Takuma Higurashi, Takashi Jono, Taisuke Akimoto, Atsushi Nakajima, Tetsuya Yamamoto, Shin Maeda, Takeshi Kaneko, Ichiro Takeuchi.

**Writing – original draft:** Shigeta Miyake.

**Writing – review & editing:** Takuma Higurashi.

## Supplementary Material

Supplemental Digital Content
