# Supplementary material for: Real-world evaluation of a computed tomography-first triage strategy for suspected Coronavirus disease 2019 in outpatients in Japan: An observational cohort study
Source: Medicine (Baltimore). 2021 Jun 4;100(22):e26161. doi: 10.1097/MD.0000000000026161 (PMC8183760; doi:10.1097/MD.0000000000026161)
Supplement: Supplemental Digital Content [file medi-100-e26161-s001.docx]

Supplemental material

Case 1


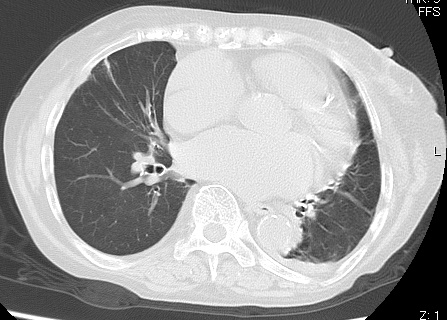

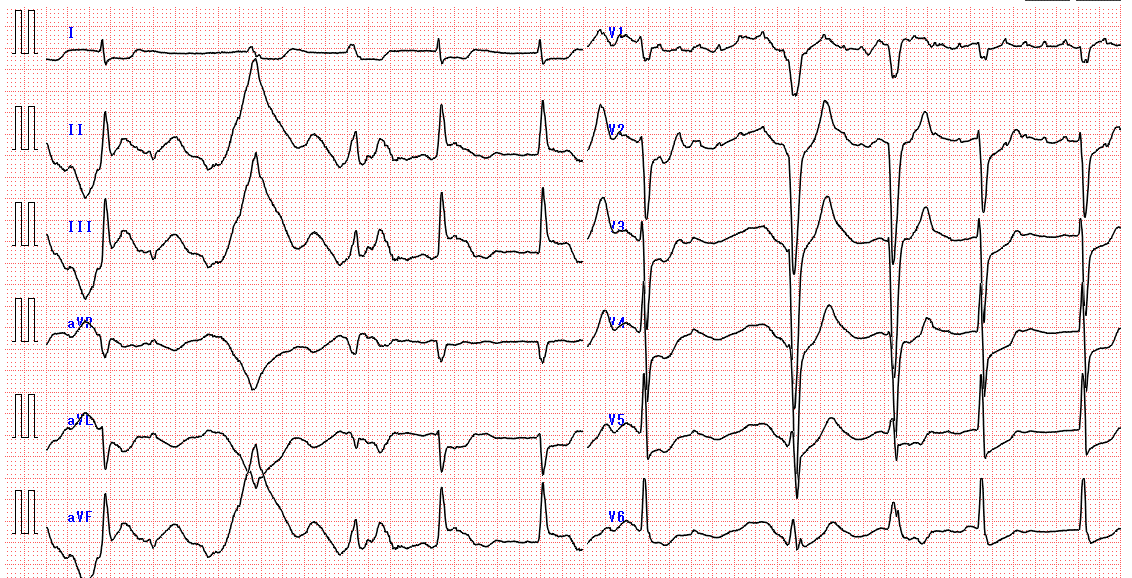

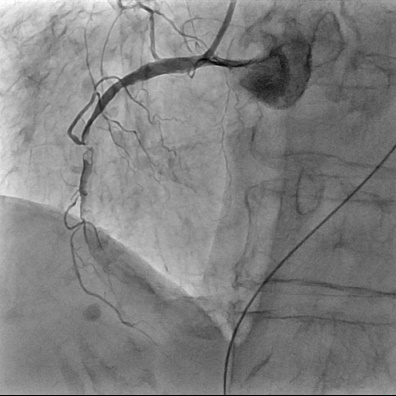


A

B

C

Case 1: Acute myocardial infarction

An 83-year-old woman presented with dyspnea and low-grade fever. Her blood pressure was 76/60 mmHg and she had signs of peripheral circulation failure on arrival. She was immediately resuscitated with bolus infusion and had a diagnostic chest CT scan (A). Chest CT showed an enlarged heart and slight pleural effusion, categorized as CO-RADS 2. The patient was included in the “COVID-19 less likely group”. After classification, she was treated according to the protocol for patients with cardiogenic shock. She received an electrocardiogram and echocardiogram. Acute myocardial infarction was diagnosed with ST elevation in II, III, and aVf on electrocardiogram (B) and asynergy of the inferior wall of the heart on echocardiogram. Finally, she received percutaneous coronary intervention (C). Despite intensive medical care, she died of myocardial infarction.

Case 2

B

A

| The Cerebrospinal fluid findings | |
| --- | --- |
| Cell counts | 448 cells / 3 visual fields |
| White blood cells | 20 cells / 1 visual field |
| Glucose | 57 mg/dL |


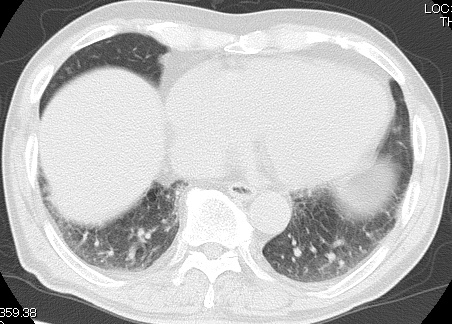


Case 2: Meningitis

A 76-year-old man presented with fever and fatigue. He had a medical history of herbal medicine-induced interstitial pneumonia. His body temperature was 39.3°C, his oxygen saturation (SpO2) was 98% on room air upon arrival. Chest CT scan showed no suspected features of COVID-19 and was categorized as CO-RADS 1 (A). He was included in the “COVID-19 suspected group” because we could not rule out COVID-19 owing to his strong fatigue and high fever, and the patient was at high risk because of his medical history. After admission, a detailed fever work-up was performed, including examination of cerebrospinal fluid (CSF). His CSF showed elevated cell counts and decreased glucose (B). The patient was diagnosed with meningitis and immediately treated with antibiotics and corticosteroids. He tested negative for SARS-CoV-2 on PCR, but the test result was only available after he had been diagnosed with meningitis.

Case 3


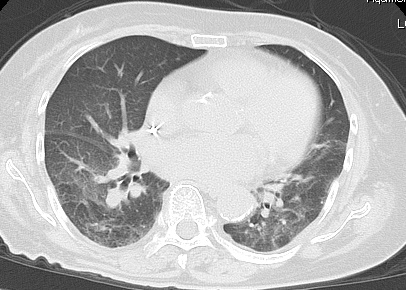

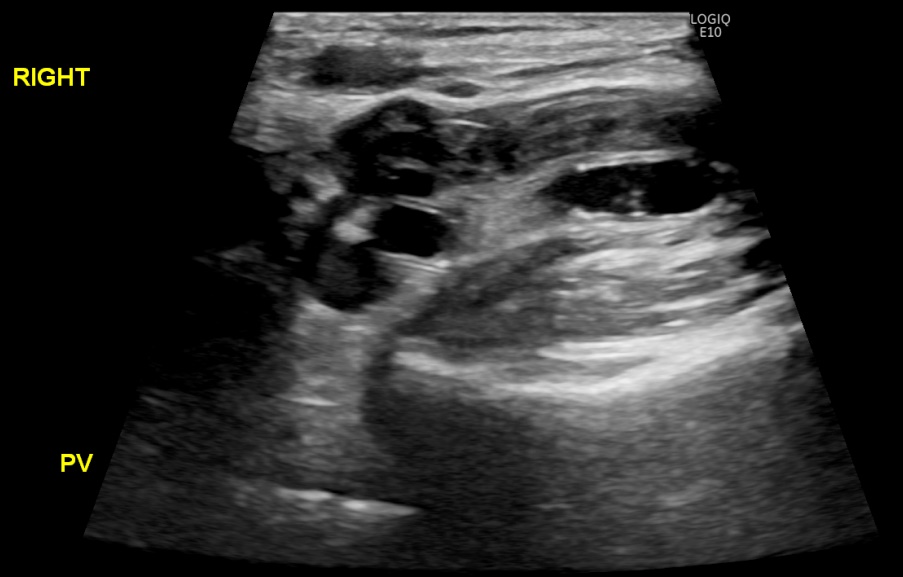


A

B

Case 3: Pneumonia with venous thrombosis

An 87-year-old woman presented with fever and dyspnea. Her body temperature was 39.4°C and her SpO2 was 84% on room air upon arrival. Her chest CT showed bilateral ground-glass opacities and was categorized as CO-RADS 4 (A). She was included in the “COVID-19 suspected group”. Her blood chemistry tests revealed slight inflammation and elevated D-dimer. The patient was hospitalized because she needed oxygen for respiratory support. Her SARS-CoV-2 PCR test result was determined to be negative; she was then examined using lower limb ultrasound (B, red arrow). These investigations revealed massive deep venous thrombosis.
